# Ultrasmall Peptides Self-Assemble into Diverse Nanostructures: Morphological Evaluation and Potential Implications

**DOI:** 10.3390/ijms12095736

**Published:** 2011-09-07

**Authors:** Anupama Lakshmanan, Charlotte A.E. Hauser

**Affiliations:** Institute of Bioengineering and Nanotechnology, 31 Biopolis Way, The Nanos 138669, Singapore; E-Mail: alakshmanan@ibn.a-star.edu.sg

**Keywords:** ultrasmall peptides, self-assembly, bioengineering, nanotechnology, supramolecular structures, origin of life

## Abstract

In this study, we perform a morphological evaluation of the diverse nanostructures formed by varying concentration and amino acid sequence of a unique class of ultrasmall self-assembling peptides. We modified these peptides by replacing the aliphatic amino acid at the *C*-aliphatic terminus with different aromatic amino acids. We tracked the effect of introducing aromatic residues on self-assembly and morphology of resulting nanostructures. Whereas aliphatic peptides formed long, helical fibers that entangle into meshes and entrap >99.9% water, the modified peptides contrastingly formed short, straight fibers with a flat morphology. No helical fibers were observed for the modified peptides. For the aliphatic peptides at low concentrations, different supramolecular assemblies such as hollow nanospheres and membrane blebs were found. Since the ultrasmall peptides are made of simple, aliphatic amino acids, considered to have existed in the primordial soup, study of these supramolecular assemblies could be relevant to understanding chemical evolution leading to the origin of life on Earth. In particular, we propose a variety of potential applications in bioengineering and nanotechnology for the diverse self-assembled nanostructures.

## 1. Introduction

Self-assembly of biomolecules is exploited not only by nature for biological evolution and speciation, but also in modern day bioengineering and nanotechnology. It enables the creation of a diverse range of hierarchical nanostructures [1–4]. Self-assembling peptides are especially relevant for biomedical applications due to their close resemblance to natural polypeptides. They can organize into different structures such as membranes, fibers, films, tapes, micelles, tubes, needles, rods and spheres [3,5–9]. Their biocompatibility, along with tunable physical and chemical properties, make peptide nanostructures suitable for applications in tissue engineering, regenerative medicine, drug delivery and electronic devices for bio-sensing, diagnosis and drug development [10,11].

Recently, we created a unique class of natural tri- to heptapeptides made of simple, non-aromatic amino acids that self-assemble in water to form hydrogels [12,13]. This assembly involves a conformational transition of the structurally unorganized monomers into metastable α-helical intermediates that terminate in cross-β structures [12]. The peptides have a characteristic sequence motif that consists of an aliphatic amino acid tail of decreasing hydrophobicity capped by a polar head, which makes them amphiphilic. The head group includes acidic, neutral or basic non-aromatic, polar amino acids. The decrease in hydrophobicity from the *N*-terminus (acetylated to suppress charge effects) to *C*-terminus strongly improves ease of self-assembly, stability and strength of the nanostructures [12]. The self-assembly of these ultrasmall peptides is initiated by molecular recognition via parallel-antiparallel pairing. This is in turn driven by a subtle interplay of non-covalent interactions, mainly hydrogen bonding and van der Waal’s interactions. In general, the contribution of different forms of non-covalent interactions to self-assembly can vary substantially, depending on the type of peptide used, and is in most cases not fully understood [14].

In our previous studies, we performed alanine scans to investigate the contribution of individual amino acid positions and changed polarity at the *C*-terminus by using acidic, basic or neutral amino acids [12–13]. The results indicated that the sequence of the peptides, length of the hydrophobic tail and polarity of the head group were critical factors affecting self-assembly. While fibers were observed with all investigated candidates, the whole range of possible nanostructures was not characterized in detail. In addition, it has been confirmed by X-ray fiber diffraction that our peptides self-assemble into amyloid structures [12]. Therefore, we wanted to investigate the effect of introducing aromatic residues on the self-assembly of this class of peptides.

In this study, we performed a morphological evaluation of different nanostructures produced by the aliphatic peptides at low and high concentrations. In addition, the best performing hexamer with respect to propensity of gelation and gel strength, namely LIVAGD and the smallest trimer peptide IVD, were modified by replacing the aspartic acid residue at the *C*-terminus with an aromatic amino acid (F, W and Y). Morphological and structural evaluations were carried out using Field Emission Scanning Electron Microscopy (FESEM) to examine the diverse range of self-assembled structures formed by modified and unmodified ultrasmall peptides. We will discuss the potential applications of these nanostructures in bioengineering and nanotechnology. In the light of seminal experiments by Oparin and Haldane [15], the amino acid sequences used in our ultrasmall aliphatic peptides may also be relevant for understanding the origin the life.

## 2. Results and Discussion

### 2.1. Nanostructures Formed at Low Concentrations of Aliphatic Peptides

**Figure 1:**
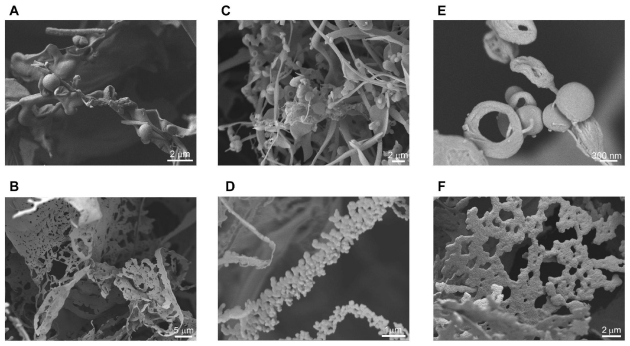
Morphological characterization of the supramolecular structures formed by aliphatic ultrasmall peptides at low concentrations (close to or far below the minimum gelation concentration) using FESEM. Representative images of hollow nanospheres and blebs formed by (**A**) Ac-ID_3_ (L) (5 mg/mL) at 8000×; (**B**) Ac-ID_3_ (L) (2 mg/mL) at 2200×; (**C**, **D**) Ac-LD_6_ (L) (0.1 mg/mL) at 4000× and 15,000× respectively; (**E**) Ac-AD_6_ (L) (5 mg/mL) at 37,000×; (**F**) Ac-LD_6_ (L) (1.3 mg/mL) at 6000×.

In order to examine the morphology of nanostructures formed by the self-assembling peptides listed in Table 1, structural evaluation was performed using low-temperature FESEM. Previously, we had reported a critical gelation concentration for the peptides to form hydrogels [13]. Nonetheless, we observed hollow nanospheres and structures resembling membrane blebs already at concentrations far below that required for gelation (Figure 1). For ID_3_, membrane structures and hollow nanospheres were observed at concentrations of 2 and 5 mg/mL respectively, far below the critical gelation concentration of 10 mg/mL (Figure 1A,B). At this concentration, no gelation or fiber networks were seen. For LD_6_, hollow nanospheres and membranous blebs were seen at a low peptide concentration of 0.1 mg/mL (Figure 1C,D), around 10 times lower than that required for gelation. Membrane structures were also observed for LD_6_ and AD_6_ at concentrations near the critical gelation concentration (Figure 1F). For AD_6_, hollow nanospheres were seen at 5 mg/mL, close to the critical concentration (Figure 1E). The hollow nanospheres (Figure 1E) seem to be composed of condensed fibers. The factors that drive further organization of assembled fibers into nanospheres and meshes are not yet clear. It is likely that the process is controlled by specific thermodynamic parameters. The nanospheres can be considered equivalent to the hydrogel cavities that are formed at higher concentrations. The nanospheres formed by these amphiphilic self-assembling peptides at low concentrations have potential applications as natural vehicles or carriers for active and passive drug delivery.

The first formulation of a theory on a strictly materialistic basis for the origin of life was Haldane’s and Oparin’s hypothesis of a primordial soup, containing all essential chemicals required to form biomolecules of increasing complexity by a process of chemical evolution [15]. Miller demonstrated that amino acids can be formed in a model atmosphere containing methane, hydrogen, ammonia, water and some other simple compounds under the influence of electric discharges and heat [16]. Miller’s work laid the foundation for exploring pre-biotic chemistry by using controlled model environments [17]. Many of these gas-phase experiments as reviewed by Rode *et al*. point to the existence of simple, aliphatic amino acids such as leucine (L), glycine (G), valine (V), alanine (A), aspartic acid (D) and glutamic acid (E) in the primordial soup [17,18]. The Salt Induced Peptide Formation (SIPF) reaction in combination with adsorption processes on clay minerals provides one of the simplest and most universal mechanisms for production of the first peptides on Earth [17]. After SIPF reaction times of a few days, heptapeptides were the largest sub-units analyzed [19]. This makes our study of self-assembling ultrasmall peptides, made of simple aliphatic residues, such as LIVAGD, AIVAGD and IVD, especially important in the context of better understanding prebiotic chemical evolution. Hollow spheres and membrane blebs formed by these amphiphilic ultrasmall peptides (Figure 1A,C–E) can have diameters between a few hundred nanometers to a micrometer. These self-assembled supramolecular structures may have very likely played an important role in biological evolution and speciation by providing specialized niches or microenvironments. The acidic pH and high salt concentration of the primordial soup may have caused immediate degradation of oligonucleotides and hydrolysis of sensitive compounds [17]. In this regard, self-assembled peptide nanostructures that are more stable under these conditions might have been one of the first cell-like structures protecting and encapsulating more sensitive molecules like RNA/DNA from the hostile environment of primitive Earth.

### 2.2. Long, Helical Fibers Formed by Aliphatic Peptides

**Figure 2:**
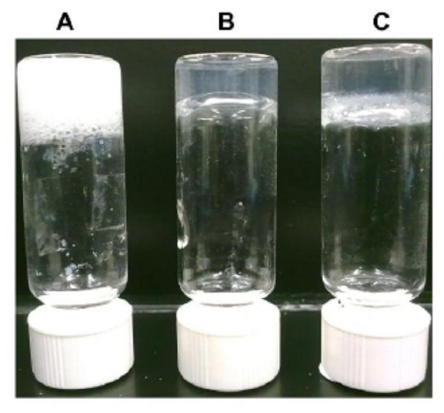
Representative pictures of hydrogels formed by self-assembling peptides containing aliphatic amino acids. Inverted vials show the formation of solid, stable hydrogels (**A**) Ac-ID_3_ (L) 14 mg/mL; (**B**) Ac-AD_6_ (L) 5 mg/mL; (**C**) Ac-LD_6_ (L) 2.5 mg/mL.

**Figure 3:**
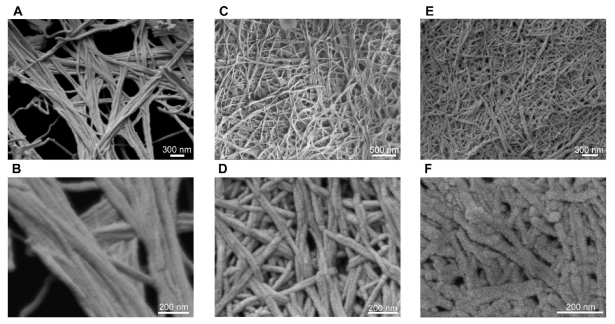
Morphological characterization of the supramolecular networks formed by aliphatic ultrasmall peptides using FESEM. Representative images of fibers formed by (**A**, **B**) Ac-ID_3_ (L) (15 mg/mL) at 30,000× and 100,000× respectively; (**C**, **D**) Ac-AD_6_ (L) (5 mg/mL) at 30,000× and 95,000× respectively; (**E**) Ac-LD_6_ (L) (10 mg/mL) at 35,000×; (**F**) Ac-LD_6_ (L) (2 mg/mL) at 150,000×.

Our unique class of ultrasmall aliphatic peptides forms hydrogels when dissolved in water at higher concentrations. The opacity and mechanical stiffness of the hydrogels increase with increasing peptide concentration [13]. Near the critical gelation concentration, clear gels were formed within hours (Figure 2B,C). At higher concentrations, gelation occured within minutes, giving translucent white gels with higher mechanical stiffness (Figure 2A). The hydrogels were shock frozen, lyophilized and observed under FESEM. Dense, interconnected networks of long, twisted helical fibers were observed (Figure 3). These long fibers entangle together to create meshes that can entrap water and form hydrogels. Majority of water molecules surround the peptide fibers (hydrophilic exterior) and are not retained within them (hydrophobic core) [12]. These supramolecular assemblies can be considered β-turn rather than β-sheet structures due to the helical turns and twists of the fibers. This was confirmed by CD spectroscopy and X-ray fiber diffraction [12]. Each helical turn in an α-helix corresponds to 3.6 amino acids and defined α-helices are formed in water by cyclic pentapeptides [20]. Thus, there is a general view that linear tri- to heptapeptides are restricted by size to form helical structures in aqueous solutions. In this context, it is interesting that even the trimers aggregate into fibers with helical twists and turns. We want to emphasize that our self-assembling peptides were not specifically designed to form α-helices. For instance, this is unlike the 28-residue peptides developed by Woolfson *et al*. with a coiled-coil heptad sequence repeat that form hydrogels through longitudinal stacking of α-helical dimers [21]. The self-assembly of our peptides involves distinct conformational transitions from random coil to anti-parallel, helical dimer pairs, which then stack on top of each other to form fibers [12]. The emergence of these supramolecular structures relies on the efficiency of conformational transitions, which are in turn dictated by the amino acid sequence.

Recent studies on the physical properties of hydrogels formed by these aliphatic peptides demonstrated their temperature-resistance and high mechanical stiffness that can be tuned by changing the pH, salt and peptide concentration [13]. In addition, the chemical composition can be modified through surface functionalization for conjugation of growth factors, matrix metalloproteinase cleavable sequences and cell adhesion factors [22,23]. The biocompatibility of the peptide hydrogels with different types of cells has also been demonstrated [13]. Thus, the peptide hydrogels can be used to make functional 3-D scaffolds for tissue engineering and injectable formulations for regenerative medicine.

### 2.3. Short, Straight Fibers Formed by Modified Peptides with Aromatic Amino Acids

**Figure 4:**
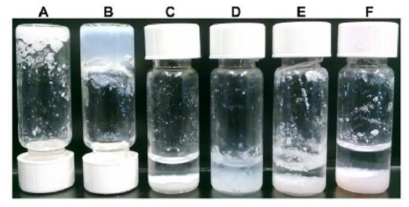
Representative pictures of hydrogels/aggregates formed by self-assembling peptides containing an aromatic amino acid at the *C*-terminus. Inverted vials show the formation of solid, stable hydrogels. (**A**) Ac-LF_6_ (L) 10 mg/mL; (**B**) Ac-IF_3_ (L) 5 mg/mL; (**C**) Ac-LY_6_ (L) 10 mg/mL; (**D**) Ac-IY_3_ (L) 5 mg/mL; (**E**) Ac-LW_6_ (L) 10 mg/mL; (**F**) Ac-IW_3_ (L) 5 mg/mL after 72 h.

**Figure 5:**
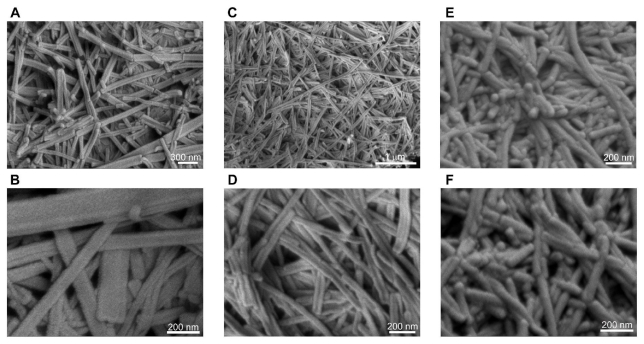
Morphological characterization of the supramolecular networks formed by ultrasmall peptides containing an aromatic amino acid as head group using FESEM. Representative images of fibers formed by (**A**, **B**) Ac-LW_6_ (L) (10 mg/mL) at 40,000× and 100,000× respectively; (**C**, **D**) Ac-IY_3_ (L) (10 mg/mL) at 25,000× and 80,000× respectively; (**E**, **F**) Ac-IF_3_ (L) (5 mg/mL) at 80,000× and 100,000× respectively.

Design of the peptide sequence holds the cues required for non-covalent interactions that drive molecular recognition and self-assembly. Hence, it is necessary to explore the effect of sequence modification on self-assembly and supramolecular organization of this unique class of peptides. This was done by replacing the aspartic acid residue of LD_6_ and ID_3_ at the polar *C*-terminus with an aromatic amino acid such as tyrosine, phenylalanine, tryptophan. This modification had a marked influence on the ease of self-assembly, the gelation capacity and the morphology of the fibers (Figure 4,5). Of the six different sequences with aromatic residues, only those with phenylalanine formed relatively stable hydrogels (Figure 4A,B). IW_3_ formed a weak hydrogel that collapsed into smaller aggregates in aqueous solution within 72 h of formation (Figure 4F). The remaining three sequences, namely IY_3_, LY_6_ and LW_6_ only formed small aggregates in solution (Figure 4C–E). These results can be attributed to the formation of short, straight fibers by the modified peptides (Figure 5). The short and flat fibers are unable to entangle and make dense meshes required to effectively entrap water to produce stable hydrogels. This is due to their inability to bend and curl. Some peptides formed straight fibers with a flat morphology (Figure 5A–D). These flat nanostructures are similar to assemblies observed for fully aromatic di-tripeptides such as FF and FFF [24]. These assemblies can be regarded as flat β-sheets rather than β-turns observed for the aliphatic sequences.

The higher stability of hydrogels formed by phenylalanine-containing peptides compared to the other modified sequences can be explained by considering the chemical structure of different aromatic amino acid residues. The lone pair of electrons on the nitrogen (in tryptophan) and oxygen (in tyrosine) can interact with the π-electrons of the aromatic ring, giving resonance structures. The same cannot be said for the phenylalanine residue, which cannot form resonance structures. Also, IF_3_ forms more rounded, cylindrical fibers compared to the flat fibers formed by the other sequences (Figure 5E–F). The round fibers probably form a better mesh compared to the flat fibers. In addition, a cylindrical fiber has much greater total surface area than a cuboidal fiber of comparable dimensions. This increases the water retention capacity for the cylindrical fibers as a greater area of the hydrophilic exterior is in contact with the water molecules.

The occurrence of aromatic amino acids in naturally occurring motifs such as amyloidogenic sequences has led to the general view that these residues are essential for molecular recognition and self-assembly [25]. The exact mechanism for aromatic stabilization in self-assembly is unclear and proposed theories include π-stacking or hydrophobic interactions among aromatic residues. T-shaped stacking of the aromatic rings, with the planar surfaces roughly perpendicular to each other has also been reported [24,26,27]. Furthermore, it has been suggested that aromatic amino acids are important for self-assembly due to their increased hydrophobicity and β-sheet propensity rather their aromaticity [28]. Compared to our aliphatic peptides, the modified aromatic sequences form straight fibers without helical turns or twists. The introduction of a planar aromatic ring at the *C*-terminal head introduces a certain degree of steric hindrance that prevents the formation of helical turns in the fibers. This steric effect is a possible explanation for the lack of long, extended assemblies for these modified peptides. Furthermore, crystallographic work done by Gorbitz *et al*. on the FF peptide suggests that the assembled nanotubes may be structurally similar to FF crystals [26]. On the other hand, Gazit *et al*. concluded that the crystal packing of the peptide represented a completely different molecular arrangement compared to the individual self-assembled nanotubes [29]. More investigations clearly need to be conducted in order to determine the exact mechanism by which aromatic residues interact and influence self-assembly.

The short, straight nanostructures produced by self-assembly of modified peptide sequences can provide the building blocks for development of modern nanodevices. For example, Gazit *et al*. have demonstrated the use of l-diphenylalanine peptide nanotubes as degradable molds for casting silver nanowires [29]. These nanotubes have also been deposited on the surface of simple screen-printed electrodes, significantly enhancing their sensitivity [30]. The remarkable improvement caused by nanotube deposition was linked to an increase in the functional electrode surface area and enhanced electrical conductivity due to electron transfer between spatially aligned aromatic assemblies [30]. Attachment of peptide nanotubes to gold electrodes has been used to design a precise and sensitive amperometric biosensor for determination of glucose and ethanol [31]. Thus, desirable properties of self-assembled peptide nanostructures, such as their electrocatalytic activity, surface attachment and molecular recognition abilities could pave the way to the next generation of unique biosensing and diagnostic nanodevices.

## 3. Experimental Section

Preparation of peptide solutions. All peptides (GL Biochem, Shanghai, China, ≥98% purity) were freshly prepared in order to avoid premature peptide aggregation. The peptides were acetylated at the *N*-terminus to suppress the effects of end charges. The peptides were dissolved in milliQ water by vortexing and left at room temperature to form hydrogels. Depending on the peptide concentration and the peptide sequence, the self-assembly process occurred immediately, within hours or even a day (experimental time frame for gelation). If an accelerated, hence forced assembly was needed, the peptide solution was subjected to sonication in a water bath (Barnstead Labline 9319 UltrasonicLC60H). For our model peptides (LD_6_, AD_6_ and ID_3_), no significant structural differences were observed between hydrogels produced via self-assembly and those whose assembly was forced by sonication [12,13].

FESEM studies. Samples were shock frozen in liquid nitrogen and kept at −80 °C. Frozen samples were then freeze-dried. Lyophilized samples were fixed onto a metal sample holder using a double-sided, conductive carbon tape and sputtered with platinum from both the top and the sides in a JEOL JFC-1600 High Resolution Sputter Coater. The coating current used was 20 mA and the process lasted for 50 s. The surface of interest was then examined with a JEOL JSM-7400F Field Emission Scanning Electron Microscopy system using an accelerating voltage of 2–3 kV at different magnifications from 1000× to 150,000×.

## 4. Conclusions

We have observed that peptides within the range of 3 to 7 amino acids can self-assemble into a surprisingly wide variety of nanostructures. This includes hollow nanospheres, membrane blebs, elongated helical fibers and short, flat fibers. The final supramolecular assembly is influenced by many factors such as the amino acid sequence, hydrophobicity profile and concentration. The spontaneous assembly of this class of simple peptides could provide important clues for the origin of life and lay the building blocks for the next generation of biomaterials and nanodevices.

## Figures and Tables

**Table 1:** Summary of self-assembling peptides used in this study, which exhibit defined supramolecular structures (all sequences were acetylated at the *N*-terminus).

Ultrasmall peptides containing only aliphatic amino acids		Ultrasmall peptides containing an aromatic head group	

Hexamer	Trimer	Hexamer	Trimer
LIVAGD (LD_6_)	IVD (ID_3_)	LIVAGF (LF_6_)	IVF (IF_3_)
AIVAGD (AD_6_)		LIVAGW (LW_6_)	IVW (IW_3_)
		LIVAGY (LY_6_)	IVY (IY_3_)
